# An Overview about New Methods in Management of Gag Reflex during Dental Treatment: A Systematic Review

**DOI:** 10.30476/dentjods.2022.96360.1934

**Published:** 2023-12-01

**Authors:** Mohammad Mehdizadeh, Abolfazl Mohammadbeigi, Alireza Sharifinejad

**Affiliations:** 1 Dept. of Oral and Maxillofacial Surgery, School of Dentistry, Qom University of Medical Sciences , Qom, Iran; 2 Dept. of Biostatistics and Epidemiology, School of Health, Qom University of Medical Sciences, Qom, Iran; 3 Postgraduate Student, Dept. of Pediatric Dentistry, School of Dentistry, Shiraz University of Medical Sciences, Shiraz, Iran

**Keywords:** Gagging, Gag Reflex, Dentistry, Review

## Abstract

**Statement of the Problem:**

Management of gag reflex is a challenging process during many dental treatments. Various studies have been carried out to evaluate different pharmacological and non-pharmacological techniques to control gagging.

**Purpose:**

The aim of this study is to review the available evidence on methods proposed for managing the gag reflex.

**Materials and Method:**

This systematic review adheres to the preferred reporting items for systematic review and meta-analysis (PRISMA) guidelines. A comprehensive search was conducted in English and Persian based on articles published from 2015 to 2022 (February) in PubMed, Scopus, Science Direct, Web of Science, Google Scholar, ISC and SID. All studies were first screened based on their title and abstract. The quality assessment of articles was carried out by two independent authors. Then, risk of bias evaluation was conducted according to Cochrane parameters.

**Results:**

In total, 1704 studies were identified via search. After reviewing title and abstract, 16 studies found eligible based on inclusion and exclusion criteria. Following quality and risk of bias assessment, 9 studies included in the systematic review.

**Conclusion:**

Based on the finding of this review, distraction techniques, nitrous oxide, and low-level laser therapy were found effective in management of gag reflex. The dentist should consider gag reflex management based on the type of dental treatment, gag severity, patient's age, and available capabilities.

## Introduction

The Gag reflex is an innate response of human body that protects the respiratory and gastrointestinal systems against external stimuli [ 1 ]. Stimulation of sensory receptors in the posterior area of the mouth and oropharynx causes signals across the cranial nerves 5, 9, and 10, triggering a reflex. Often, the response manifests as spasmodic muscular contractions, which are essentially an attempt to expel an external stimulus [ 2 ]. The gag reflex severity and the stimulus that initiates it vary significantly across different individuals and over time [ 3 ]. Although the gag reflex is present in the majority of people, the severe form is only experienced in a few. There is a statistically significant association between gag reflex during dental treatment and feminine gender, poor educational level, and dental anxiety [ 4 ]. It becomes less severe with age, especially after the age of four, as the child's chewing, swallowing, and breathing capacities develops [ 5 ].

Dental treatment is more difficult in people who suffer from severe gag reflex. Numerous dental procedures, including dental impression, third molar extraction, endodontic therapy, and intraoral radiography of the posterior teeth, might elicit the gag reflex [ 6 ]. Additionally, gagging might complicate some diagnostic and medical procedures, such as endoscopy [ 7 ]. Gag reflex can be induced by a variety of stimuli, including sonic vibration created by rotational devices, the smell, and taste of dental materials, direct physical stimulation of the posterior parts of the mouth, viewing of equipment, and in some circumstances, even envisioning dental treatment [ 6 ]. The neuronal connections between the gag reflex center and the cerebral cortex explain phenomena such as inducing gag with mental images and relieving gag reflex by diverting the patient's attention [ 8 ].

Numerous approaches for managing the gag reflex during dental treatment have been developed. Gag reflex management strategies are generally classed as pharmacological or non-pharmacological [ 9 ]. Local anesthetic, general anesthesia, sedatives, and herbal medications are examples of pharmacological approaches. Non-pharmacological techniques such as behavioral therapy, hypnosis, acupuncture, and laser therapy are also mentioned [ 10 ]. Numerous studies have used different indexes for quantification of the severity of gag reflex, including the gag severity index, the gag prevention index, the gag problem assessment, and the visual analogous scale as well as measuring the depth of swap penetration into the soft palate [ 11 - 14 ].

Despite the conduction of several researches, there is currently no reliable clinical guidance to assist dentists in making clinical decisions in situations of gag reflex. Most systematic reviews on this subject have been done before 2015 including a review in the Cochrane database of systematic reviews conducted in 2015, which evaluated only one eligible study for the final review [ 6 ]. This review has been updated in 2019, however, still has evaluated little evidence on this subject. Several clinical trials have done from that time and have introduced new methods in their research. These trials justify the need for new systematic reviews. 

The purpose of this study was to conduct a systematic review of the available evidence, evaluating new methods proposed for managing the gag reflex during dental treatment so that it could be used as a clinical guide during dental treatment.

## Materials and Method

The current study adheres to the preferred reporting items for systematic reviews and meta-analyses (PRISMA) guidelines published in 2020 [ 15 ]. The study was aimed to find the randomized clinical trials, which have been published between 2015 till 2022(February) and evaluated the effect of an intervention on the severity of gag reflex during dental treatment (according to 4W question method). At the first step, we developed a protocol for conducting the systematic review. The protocol included databases and search strategy, screening techniques, inclusion and exclusion criteria, data extraction, and assessment techniques.

### Search strategy

Articles published since 2015 to 2022 (February) were searched in electronic databases such as PubMed, Scopus, Science Direct, Web of Science, Google Scholar, ISC, and SID. The search was undertaken in both Persian and English. To avoid publication bias, an attempt was made to evaluate papers, dissertations, and projects, both print and non-print sources (ProQuest dissertations and theses, Irandoc dissertations). Medical subheadings (MeSH) were used to incorporate certain keywords, which are prevalent in medical papers. The key words included gag reflex, gagging, and the truncation of and . The words: glucose amino glycan and genes were excluded from the search, because in some studies the term “gag” was used as an abbreviation form of the above words.

In all, 1704 studies were included in the screening step. Then, papers were screened based on their title and abstract according to the following inclusion and exclusion criteria.

The inclusion criteria were defined as randomized clinical trials with a control group in which participants did not have a specific systemic disease or were not using a specific medicine that may impact the intensity of the gag. This study made no distinction between age groups, genders, or races, and all comparable studies were evaluated.

The exclusion criteria were defined as non-clinical or non-randomized research and studies in which individuals had a specific systemic disease or mental handicap were excluded.

### Initial screening

At this step, after deleting duplicates, titles and abstracts of all studies were reviewed. Following initial screening 16 related studies were identified eligible from the 1704 articles. Selected records were entered into Endnote software (Thomson Reuters, New York, USA). Two independent reviewers carried out initial screening. A total of 15 out of the 16 studies listed above were journal articles, while 1 was a dissertation. Additionally, 11 articles were written in English and 5 in Persian. The complete text of these articles was prepared for further assessment. To obtain all of the information necessary to evaluate the studies, the authors of some studies were contacted via e-mail during the evaluation process.

### Data extraction and quality assessment

The checklists for each research comprised information of the eligible studies using the PICOS criteria (population, intervention, comparison, outcome, study) [ 16 ]. To minimize bias in the research evaluation, the 16 papers were numbered sequentially according to their publication year. Each study's title, first author's name, and year of publication were recorded. Moreover, the e-mail address of the corresponding author was written in the checklist of each article for additional information. Following that, two authors assessed each study independently. If there was a disagreement over assessment, the third author was consulted. 

**Figure 1:**
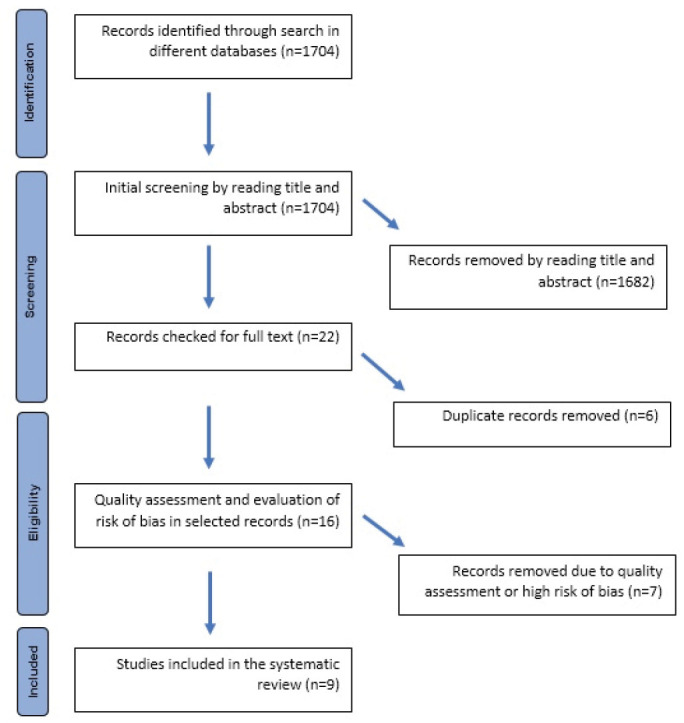
Flow diagram of search and selection of studies in the systematic review

The Cochran checklist was used to evaluate the risk of bias in any qualifying article [ 17 ]. Each article was evaluated for possible bias in case selection, randomization, blinding, and reporting of results using Cochran's criteria, and the risk of bias was classified into three categories: low risk, high risk, and unclear. Finally, a critical review of qualified articles was conducted, and conclusions were drawn from various gag reflex management techniques. The process of searching and selecting literature is illustrated in Figure 1.

Table 1 summarizes data of 16 studies in the quality assessment stage. Out of 16 eligible articles, in eight articles, the maxillary impression was made using alginate [ 18 - 25 ]. Maxillary and mandibular alginate impression was taken in two studies [ 26 - 27 ]. The other two studies examined the periapical radiographs of patients, one from maxillary teeth [ 28 ] and the other from the mandible [ 29 ]. One research examined the intensity of gag experienced during various dental surgical procedures performed under general anesthesia [ 30 ]. The type of dental therapy was not specified in one research [ 31 ]. The remaining two studies examined the degree of gag reflex following oral examination [ 32 - 33 ].

**Table 1:** Demographic specifications of studies in the first screening stage

Row	Author, year	Age	Gender	Dental treatment	Intervention	Included?*
1	Mustafa *et al*. [ 18 ] 2021	25.6	M: 24%	Maxillary impression	Distraction technique music	N
			F: 76%			
2	Kulkarni *et al*. [ 26 ] 2021	5-12	NM	Maxillary and mandibular impression	Distraction technique Colored game	Y
3	Dixit *et al*. [ 19 ] 2020	5-10	NM	Maxillary impression	Distraction technique Colored puzzle	Y
4	Jawdekar *et al*. [ 20 ] 2020	6-12	M: 50%	Maxillary impression	Ear plug and temporal tap	Y
			F: 50%			
5	Shin *et al*. [ 30 ] 2020	15-74	NM	NM	IV sedation Propofol	N
6	Balouch *et al*. [ 21 ] 2020	25-56	M: 46%	Maxillary impression	Metoclopramide	N
			F: 54%			
7	Yamamoto *et al*. [ 31 ] 2018	17-70	M: 62%	NM	IV sedation Propofol and midazolam	N
			F: 38%			
8	Debs *et al*. [ 22 ] 2017	5-11	M: 52%	Maxillary impression	Distraction technique Colored game	Y
			F: 48%			
9	Goel *et al*. [ 23 ] 2017	4-14	M: 42%	Maxillary impression	Low-level laser therapy	Y
			F: 58%			
10	Kamran *et al*. [ 27 ] 2016	21.6	M: 50%	Maxillary and mandibular impression	Adding Lidocaine to impression material	N
			F: 50%			
11	Elbay *et al*. [ 28 ] 2016	6-12	M: 68%	Radiography from maxilla	Low-level laser therapy	Y
			F: 32%			
12	Veaux *et al*. [ 29 ] 2016	14-42	M: 50%	Radiography from mandible	Nitrous oxide sedation	Y
			F: 50%			
13	Shadmehr *et al*. [ 32 ] 2016	NM	NM	Oral examination	Tannic acid patch	Y
14	Fakhrzadeh *et al*. [ 24 ] 2015	NM	M: 54%	Maxillary impression	Benzocaine topical anesthesia	N
			F: 46%			
15	Rahshenas *et al*. [ 33 ] 2015	28	M: 36%	Oral examination	Acupressure	Y
			F: 64%			
16	Ebadi *et al*. [ 25 ] 2015	24	F: 100%	Maxillary impression	Acupuncture	N

* : included in the systematic review? NM: not mentioned N: no Y: yes

Six of the sixteen studies involved children with an average age of fewer than 14 years [ 19 , 21 - 23 , 26 , 28 ]. The age distribution was not given in one paper [ 32 ]. The remaining studies were conducted on adults with a mean age of more than 20 years.

Only one research was done exclusively on females in the preceding sixteen papers [ 25 ]. Three studies did not mention the research population's gender makeup [ 19 , 26 , 32 ]. In other studies, the target group included both men and women.

### Risk of bias assessment

**Figure 2:**
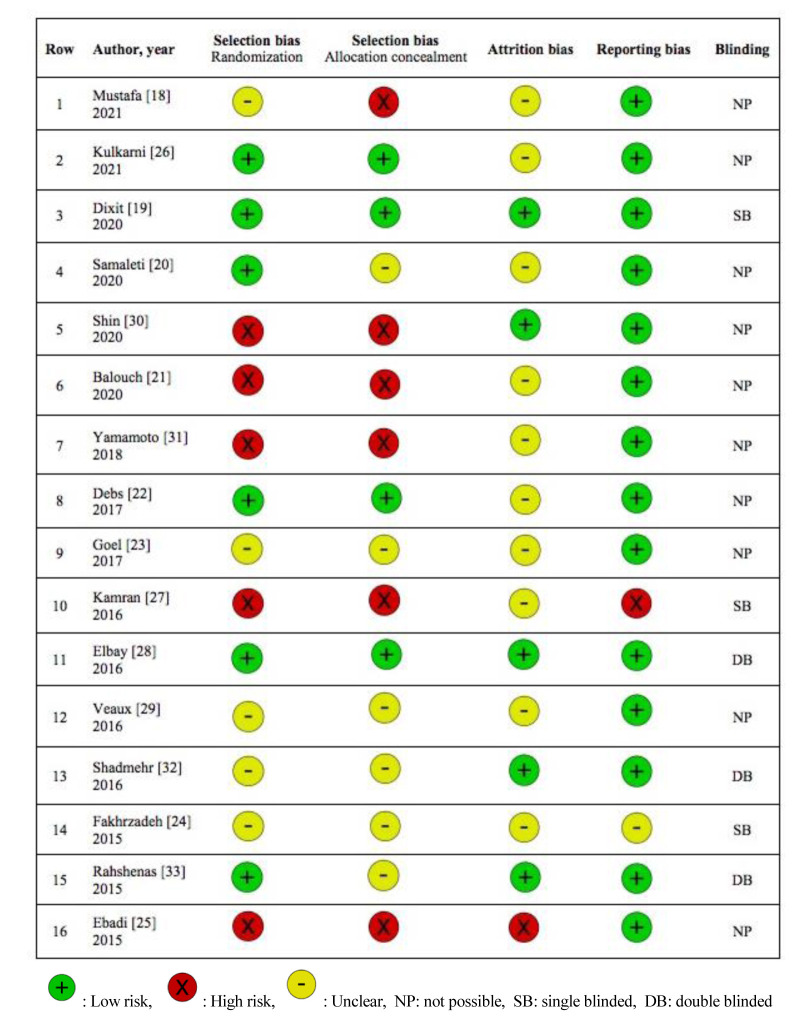
Risk of bias assessment

Figure 2 contains an overview of the risk of bias assessment. Different biases were evaluated in the present study including:

### 1. Selection bias (Randomization)

Among the evaluated studies, five were recognized as having a high probability of bias in the randomization method [ 20 , 25 , 27 , 30 - 31 ]. Only three of the sixteen researches have mentioned their method of randomization [ 19 , 21 , 28 ].

### 2. Selection bias (Allocation concealment)

Four of the studies had a high risk of bias in allocation concealment [ 18 , 20 , 30 - 31 ].

### 3. Attrition bias

In the study by Ebadi *et al*. [ 25 ] on the effects of acupuncture on gag reflex, ten participants were excluded due to their inability to bear impression. This is an illustration of attrition bias. It may be stated that the intervention's effect was overestimated positively in this study.

### 4. Reporting bias

In the study by Kamran *et al*. [ 27 ], the gag reflex severity was measured by the gag severity index before the intervention, whereas the index after the intervention was declared the gag prevention index. This incident exemplifies reporting bias. It is probable that changing the index make the findings more significant. To evaluate the intervention's effect adequately, the indices used for comparison must be identical.

### 5. Blinding

Blinding is not achievable in some experimental trials. For instance, it was impossible to blind the subject and the intervener in the research that compare the intensity of gag caused by conventional and digital impressions [ 34 ]. Only three of the papers included in this evaluation were double-blinded [ 28 , 32 - 33 ].

## Results

After quality and risk of bias assessment, nine eligible studies with low risk of bias were included in the final review, and their results were analyzed [ 19 , 21 - 23 , 26 , 28 - 29 , 32 - 33 ]. Table 2 summarizes the findings of the studies included in this systematic review.

**Table 2:** Summary of studies included in the systematic review

Row	Authors	Year	Objective	Sample Size	Statistical Analysis	Variables	Main Results
1	Kulkarni *et al*. [ 26 ]	2021	To evaluate the effect of the Intellectual colored game on the severity of gag reflex and anxiety.	50	Wilcoxon signed-rank test, Mann Whitney test	GPI	Intellectual colored game is effective in lowering gag severity and anxiety level in test group relative to the control group.
						FIS	
2	Dixit *et al*. [ 19 ]	2020	Evaluate the effect of interactive distraction method to manage gag reflex during impression.	48	SPSS, Chi square test	FIS	Interactive distraction method can effectively manage gag reflex in children.
						GISS	
						GSI	
3	Jawdekar *et al* [ 21 ]	2020	To compare earplug and temporal tap technique with distraction technique on gagging.	30	SPSS, Chi square test, Friedman test, Mann Whitney U test	GPI	Earplug and temporal tap technique did not reduce gag reflex but led to a better experience.
						5- point patient reported scale	
4	Debs *et al*. [ 22 ]	2017	To evaluate the effect of intellectual colored game on the severity of gag reflex.	41	Descriptive statistics, SPSS, Fisher’s exact test, Friedman test	GPI	There was a statistically significant decrease in GPI and FIS after intellectual color game.
						FIS	
5	Goel *et al*. [ 23 ]	2017	To determine the effect of LLLT on PC6 acupuncture point on the severity of gag reflex.	40	SPSS, Spearman correlations, Wilcoxon signed-rank test, Mann- Whitney U test	GSI	LLLT is useful in reducing anxiety level and severity of gagging. After LLLT, O2 saturation increased and pulse rate declined.
						Modified child dental anxiety scale, Pulse rate oxygen saturation	
6	Elbay *et al*. [ 28 ]	2016	To investigate the efficacy of LLLT on lowering gag reflex.	25	SPSS, McNemar test	Corah dental anxiety scale, GS score	LLLT is effective in reducing gag reflex. There was no significant correlation between gag severity and anxiety level.
7	Veaux *et al*. [ 29 ]	2016	To compare different concentrations of nitrous oxide on lowering gag reflex.	14	Wilcoxon signed-rank test, Mann Whitney test	PGS	Increasing in Nitrous oxide concentration from 30 t0 70% is effective in controlling gag. With 70% concentration, all patients having severe gag reflex could tolerate the test.
						MDAS	
						GSI	
						VAS	
8	Shadmehr *et al*. [ 32 ]	2016	To assess the tannic acid patch effect on reduction of gagging.	88	Wilcoxon signed-rank test, Mann Whitney test	Gog reflex intensity	Both statistical analyses showed significant reduction in gag severity in the test group.
9	Rahshenas *et al*. [ 33 ]	2015	To evaluate the effect of acupressure on severity of gag.	75	Wilcoxon signed-rank test, Mann Whitney test, Kruskal walis test	Glasscow scale	There was a statistically significant decrease in gag severity of case group 2 relative to the control group and case group 1(placebo).

GPI: gag prevention index; GSI: gag severity index; VAS: visual analogue scale; FIS: facial image scale; MDAS: modified dental anxiety scale

### Non-pharmacological intervention

### Mental Distraction techniques

Kulkarni *et al*. [ 26 ] assessed the effect of intellectual colored game on gag reflex. They revealed that the anxiety level was statistically lower following the game. In this article, no information was provided about the randomization method, sample size determination, and gender makeup of the population.

In the study of Dixit *et al*. [ 19 ], the effect of puzzle game on gag reflex and anxiety level was investigated.

All participants in the case group could tolerate impression, unlike those in the control group. The severity of gagging and level of anxiety were also decreased in the intervention group.

In the study of Debs *et al*. [ 22 ], gag reflex severity and anxiety level were significantly lower following intellectual colored game. A significant association between gag severity and anxiety level illustrates the fact that the child’s participation in game may boost his confidence, by releasing serotonin and endorphin [ 22 , 35 ].

### Acupressure techniques

Jawdekar *et al*. [ 21 ] evaluated the efficacy of acupressure using earplug on gag severity. According to this study, gagging was not significantly different between case and control group. It is asserted that this technique might be beneficial in suppressing gag reflex mediated by auriculotemporal nerve. However, earplug has no effect on gagging mediated by glossopharyngeal nerve, which is most responsible for gagging during dental treatments [ 36 ].

In the study of Rahshenas *et al*. [ 33 ], gag severity was compared between case group, placebo group, and no-intervention group. The reduction of gag reflex was not statistically different between placebo and non-intervention group. However, significant decrease in gag severity was stated following acupressure on palm region.

### Laser therapy

Goel *et al*. [ 23 ] assessed the effect of low-level laser therapy (LLLT) on PC6 point during impression making. Increased level of oxygen saturation and decreased gag severity and pulse rate was revealed following intervention.

Elbay *et al*. [ 28 ] evaluated the effect of LLLT on PC6 during radiography. Gag severity was significantly lower in the case group; however, the anxiety level showed no significant difference. It can be inferred that the mechanism of laser action is due to nerve stimulation and is irrelevant of anxiety level [ 37 ].

### Pharmacological intervention

### Nitrous oxide

The study of Veaux *et al*. [ 29 ] investigated the effect of nitrous oxide on gag reflex. According to their results, the severity of gag was declined relative to the increase in nitrous oxide dosage. Although 50% nitrous oxide effective in most (86%) participants, all subjects could tolerate taking radiographs by the use of 70% concentration.

### Herbal medicine

In the article of Shadmehr *et al*. [ 32 ], tannic acid patch application on the palate was resulted in gag reflex reduction. However, same reduction was noted in the placebo group. This result can be attributed to the psychological effect of the intervention. Thus, herbal medicines effect in gag reflex management is still contradictory. Other organic remedies like salt have been advocated, but there is not enough evidence to support such methods [ 36 ].

## Discussion

According to research performed during our study, the majority of approaches for controlling gag reflex during dental treatment have focused on distraction techniques and diverting the patient's attention away from the ongoing therapy (Table 1). According to the methodology of these studies, it appears that these approaches are more helpful in people with a mild to moderate gag reflex. Thus, the existing data support the efficacy of distraction methods in reducing gag reflex associated with dental treatment, particularly in children. Among different distraction techniques, intellectual games are found the most effective [ 19 , 22 , 26 ]. However, other methods like listening to music have shown lower impact on gag. In the study of Mustafa *et al*.[ 18 ], it has been shown that listening to music reduces the anxiety during dental treatment, although there is not enough evidence to verify that music reduces gag severity. It can be inferred that, playing games can better involve children attention and are more effective in mitigating gagging.

In the systematic reviews published prior to 2015, the level of evidence on gag reflex management techniques was evaluated low, and their available data did not support the efficacy of any particular therapy [ 6 , 8 , 38 ]. However, based on the findings of our review, scientific data supports the efficacy of distraction techniques, nitrous oxide, and LLLT.

The investigation on usefulness of nitrous oxide in alleviating gag reflex verifies this method's efficacy [ 29 ]. Similar findings have been observed in Chidiac *et al*. [ 39 ] research. Due to the common use of it in dentistry and the fact that it is safe and widely accepted among patients, present data support the adoption of this strategy. Additionally, this method can be used easily during routine dental procedures like taking intraoral radiographs. Whilst, other types of medical interventions like intravenous (IV) sedation sound not logical to be used routinely.

Among the high-quality evidence studies, two examined the use of LLLT on PC6 accupoint for effective gag reflex control [ 23 , 28 ]. Both above studies used diode laser on the same palm region. Goel *et al*. [ 23 ] have used laser with the power of 0.5mW, wavelength 940 nm, energy 4J, and 3-4mm away from the tissue for 1 minute. While Elbay *et al*. [ 28 ], applied laser with a continuous wavelength of 810 nm, having 1 cm distance from the target area and 4J energy density for 14 seconds. Due to the extensive usage of lasers in dental clinics, it appears that, this less invasive technology might be useful in reducing gag reflex associated with dental treatment. The study of Soltani *et al*. [ 40 ], also shows significant reduction in gag after LLLT application on the same point.

There is little evidence to support the use of local anesthetics, mouthwashes, or the addition of anesthetics to the impression material for controlling gag reflex effectively. None of the studies conducted using these approaches were determined to be of sufficient quality for final assessment. In the studies performed by Bassi *et al*. [ 8 ], and Means *et al*. [ 41 ], the use of superficial topical anesthetic has shown no discernible impact and has even recorded instances of increased gag intensity following its administration. Based on the findings of our review, applying topical anesthesia has no significant effect on reducing the gag reflex. 

Systemic medications for the suppression of the gag reflex, such as intravenous sedation, are more frequently used in patients with moderate to severe gag reflex. The available evidence on the method and dosage of sedation required for effective gag reflex management is insufficient and requires additional investigation. Now, the only accessible pharmaceutical approaches are general anesthesia and intravenous sedation, which are impractical except in the situations of specific treatments with severe gag reflex.

The results of studies on the efficacy of acupuncture and acupressure for controlling gag reflex are likewise conflicting. Based on the current available data, acupressure and laser-therapy techniques performed on PC6 accupoint on the palm region is found effective in suppressing gag reflex [ 23 , 28 , 33 , 42 ]. However, other accupoint like external auditory canal was not effective [ 21 , 43 ]. Additional controlled research is required in this area. Since the counterintuitive efficiency of these approaches has occasionally been linked to distraction effects, future research should compare the effectiveness of acupuncture to other gag reflex treatment strategies, particularly attention-diversion methods.

## Conclusion

According to the findings of this systematic review, scientific data supports the efficacy of distraction techniques, nitrous oxide, and LLLT. In general, there is still no one-size-fits-all technique for managing gag reflex during dental treatment. The dentist should manage gag reflex in accordance with the type of dental treatment, the patient's level of gag reflex, the patient's age, and available capabilities, and facilities. 

## References

[1] Ardelean L, Bortun C, Motoc M ( 2003). Gag reflex in dental practice- etiological aspects. TMJ.

[2] Conny DJ, Tedesco LA ( 1983). The gagging problem in prosthodontic treatment. Part II: Patient management. J Prosthet Dent.

[3] Wright SM ( 1981). Medical history, social habits, and individual experiences of patients who gag with dentures. J Prosthet Dent.

[4] Van-Houtem CM, Van-Wijk AJ, Boomsma DI, Ligthart L, Visscher CM, de-Jongh A ( 2015). Self-reported gagging in dentistry: prevalence, psycho-social correlates and oral health. J Oral Rehabil.

[5] Bilello G, Fregapane A ( 2014). Gag reflex control through acupuncture: a case series. Acupunct Med.

[6] Prashanti E, Sumanth KN, Renjith George P, Karanth L, Soe HH ( 2015). Management of gag reflex for patients undergoing dental treatment. Cochrane Database Syst Rev.

[7] Shin S, Kim S ( 2017). Dental treatment in patients with severe gag reflex using propofol-remifentanil intravenous sedation. J Dent Anesth Pain Med.

[8] Bassi GS, Humphris GM, Longman LP ( 2004). The etiology and management of gagging: a review of the literature. J Prosthet Dent.

[9] Hamedani S, Farshidfar N ( 2021). The predicament of gag reflex and its management in dental practice during COVID-19 outbreak. J Dent Sci.

[10] Saunders RM, Cameron J ( 1997). Psychogenic gagging: identification and treatment recommendations. Compend Contin Educ Dent.

[11] Akarslan ZZ, Biçer AZ ( 2012). Utility of the gagging problem assessment questionnaire in assessing patient sensitivity to dental treatments. J Oral Rehabil.

[12] Dickinson CM, Fiske J ( 2005). A review of gagging problems in dentistry: I. Aetiology and classification. Dent Update.

[13] Johnson EW ( 2001). Visual analog scale (VAS). Am J Phys Med Rehabil.

[14] Rosted P, Bundgaard M, Fiske J, Pedersen AM ( 2006). The use of acupuncture in controlling the gag reflex in patients requiring an upper alginate impression: an audit. Br Dent J.

[15] Page MJ, McKenzie JE, Bossuyt PM, Boutron I, Hoffmann TC, Mulrow CD, et al ( BMJ 2021). The PRISMA 2020 statement: an updated guideline for reporting systematic reviews. Systematic Reviews.

[16] Methley AM, Campbell S, Chew-Graham C, McNally R, Cheraghi-Sohi S ( 2014). PICO, PICOS and SPIDER: a comparison study of specificity and sensitivity in three search tools for qualitative systematic reviews. BMC Health Serv Res.

[17] Lasserson TJ, Thomas J, Higgins JP Starting a review. https://training.cochrane.org/handbook/current/chapter-01.

[18] Mustafa N, Ishak NH, Mohd Rosli NA, Nik Zulkifeli NR, Rajali A ( 2021). Self-preference music for gagging patient: Effect on physiology and oral health-related quality of life during dental impression. Complement Ther Clin Pract.

[19] Dixit UB, Moorthy L ( 2021). The use of interactive distraction technique to manage gagging during impression taking in children: a single-blind, randomised controlled trial. Eur Arch Paediatr Dent.

[20] Balouch F, Farahmandnia Y, Riahi P ( 2020). The effect of metoclopramide on elimination of nausea reflex during impression making. J Res Dent Sci.

[21] Jawdekar A, Samaleti S ( 2020). Comparison of “Earplug and Temporal Tap Technique” with Standard Distraction Method on Gag Reflex Related to Maxillary Impression-Making in 6 to 12-Year-Old Children: A Crossover Study. J South Asian Assoc Ped Dent.

[22] Debs NN, Aboujaoude S ( 2017). Effectiveness of Intellectual Distraction on Gagging and Anxiety Management in Children: A Prospective Clinical Study. J Int Soc Prev Community Dent.

[23] Goel H, Mathur S, Sandhu M, Jhingan P, Sachdev V ( 2017). Effect of Low-level LASER Therapy on P6 Acupoint to Control Gag Reflex in Children: A Clinical Trial. J Acupunct Meridian Stud.

[24] Farrier S, Pretty IA, Lynch CD, Addy LD ( 2011). Gagging during impression making: techniques for reduction. Dent Update.

[25] Ebadi N, Mazaheri Tehrani A, Valaii N, Fallahi F, Norouzi S ( 2014). Efficacy of Acupuncture in reduction of the Gag Reflex in Patients Requiring Upper Alginate Impression. J Res Dent Sci.

[26] Kulkarni P, Chhattani B, Agrawal N, Mali S, Kale S, Thakur NS ( 2021). Management of Gagging and Anxiety in Children by Play Way Method. Int J Drug Res Dent Sci.

[27] Kamran MF, Qamar R ( 2016). An easy and effective way to reduce gag during orthodontic impression recording. Pakistan Ortho J.

[28] Elbay M, Tak Ö, Şermet Elbay Ü, Kaya C, Eryılmaz K ( 2016). The use of low-level laser therapy for controlling the gag reflex in children during intraoral radiography. Lasers Med Sci.

[29] De-Veaux CK, Montagnese TA, Heima M, Aminoshariae A, Mickel A ( 2016). The Effect of Various Concentrations of Nitrous Oxide and Oxygen on the Hypersensitive Gag Reflex. Anesth Prog.

[30] Seok U, Ji S, Yoo S, Kim J, Kim S, Kim J ( 2016). A survey of the intravenous sedation status in one provincial dental clinic center for the disabled in Korea. J Dent Anesth Pain Med.

[31] Yamamoto T, Fujii-Abe K, Fukayama H, Kawahara H ( 2018). The Effect of adding midazolam to propofol intravenous sedation to suppress gag reflex during dental treatment. Anesth Prog.

[32] Shadmehr E, Hahshemi A, Hasheminia SM ( 2016). Evaluation of the effect of tannic acid patch on gag reflex in the areas of soft palate and tonsils. J Isfahan Dent Sch.

[33] Rahshenas N, Nasermostofi S, Valaii N, Farajzad A ( 2015). The effect of acupressure on the gag reflex. J Res Dent Sci.

[34] Burhardt L, Livas C, Kerdijk W, van-der-Meer WJ, Ren Y ( 2016). Treatment comfort, time perception, and preference for conventional and digital impression techniques: A comparative study in young patients. Am J Orthod Dentofacial Orthop.

[35] Donaldson ZR, Piel DA, Santos TL, Richardson-Jones J, Leonardo ED, Beck SG, et al ( 2014). Developmental effects of serotonin 1A autoreceptors on anxiety and social behavior. Neuropsychopharmacology.

[36] Stefos S, Zoidis P, Nimmo A ( 2019). Managing gag reflex during removable partial denture treatment: a review and a clinical report. J Prosth.

[37] Tripathi S, Mittal R, Rawat P, Koul A ( 2018). Accupuncture therapy: an emerging adjunct in prosthodontic care. Dent Adv Res.

[38] Ali S, George B, Kirmani U, Al-Saiari AKA, Almasabi FRA, Iqbal Z ( 2019). Gagging and its management in prosthodontic patients: a review of literature. Biomedica.

[39] Chidiac JJ, Chamseddine L, Bellos G ( 2001). Gagging prevention using nitrous oxide or table salt: a comparative pilot study. Int J Prosthodont.

[40] Soltani P ( 2015). Evaluation of the effects of acupuncture on P6 and anti-gagging acupoints on the gag reflex. Dental Hypotheses.

[41] Means CR, Flenniken IE ( 1970). Gagging-a problem in prosthetic dentistry. J Prosth Dent.

[42] Alkaissi A, Evertsson K, Johnsson VA, Ofenbartl L, Kalman S ( 2002). P6 acupressure may relieve nausea and vomiting after gynecological surgery: an effectiveness study in 410 women. Canadian J Anesth.

[43] Cakmak YO, Ozdogmus O, Günay Y, Gürbüzer B, Tezulaş E, Kaspar EC, et al ( 2014). An earplug technique to reduce the gag reflex during dental procedures. Forsch Komplementmed.

